# Seventeen-year outcome of surgical management of severe early onset kyphoscoliosis in a patient with arthrochalasia-type Ehlers–Danlos

**DOI:** 10.1007/s43390-025-01071-6

**Published:** 2025-04-14

**Authors:** Ronan McKeogh, Sashin Ahuja, John Howes

**Affiliations:** 1 School of Medicine, Cardiff University, Cochrane Building, Heath Park, Cardiff, CF14 4YU UK; 2 Department of Trauma and Orthopaedics, University Hospital of Wales, Cardiff, UK

**Keywords:** Early-onset scoliosis, Growth rods, aEDS, Arthrochalasia

## Abstract

We report on the surgical management of severe early onset kyphoscoliosis in a 5-year-old patient with the rare arthrochalasia subtype of Ehlers–Danlos syndrome, who we have followed for 17 years post-operatively. Successful correction of the deformity with an excellent outcome was achieved with minimal morbidity using MAGEC™ (MAGnetic Expansion Control) growth rods instead of traditional growth rods, undertaken with the close involvement of plastic surgical colleagues. Our patient suffered only one minor surgical complication (thought to be allergic rather than traumatic in origin), despite having skin so fragile that rubbing his skin with a disinfectant wipe was sufficient to cause skin breakdown. The non-invasive lengthening that the MAGEC rods allowed enabled us to avoid repeated open surgeries which would have had a high risk of complications, most notably wound breakdown with poor healing, and we advocate their use in similar cases.

## Introduction

Arthrochalasia-type (formerly known as EDS type VIIA and VIIB) Ehlers–Danlos syndrome (aEDS) occurs as a result of alterations in type 1 collagen. It can be confirmed with genetic testing, and the diagnostic criteria include congenital bilateral hip dislocation, skin hyperextensibility and fragility, severe generalized joint hypermobility [1]. There is no reliable data on the exact prevalence of aEDS.

Spinal deformity can occur in patients with some forms of EDS as early as the first year of life, with kyphoscoliosis then commonly progressing to a severe state in early childhood [2], putting such patients at risk of thoracic insufficiency syndrome. The treatment of early onset scoliosis (EOS), defined as spinal deformity before the age of 10, presents a dilemma as progression of the curvature needs to be prevented but extensive fusion can negatively impact the development of the lungs [3]. Therefore, it is typically managed with repeated surgical procedures to lengthen growth rods, to correct deformity preserving development.

However, the surgical correction of scoliosis in people with aEDS poses a unique challenge given the elevated risk of major complications due to the fragility of connective tissues. Operative complications can include devastating outcomes such as paraplegia, or fatal hemorrhage due to vascular fragility, as well as being fraught with wound complications due to poor healing and subsequent wound infection [4, 5].

## Case report

A 5-year-old boy with established aEDS with severe kyphoscoliosis was referred for a further opinion after review at two other specialist spinal centers. The diagnosis of aEDS had been previously established by a clinical geneticist.

At initial referral, he was 95 cm and 17 kg. He had a history of multiple joint dislocations and had hydrocephalus with a ventriculoperitoneal (VP) shunt in place. He had unilateral deafness. His parents reported that his skin was so fragile and friable that a simple pinch had been known to cause skin breakdown.

He mobilized using a wheelchair but could also crawl.

**Fig. 1:**
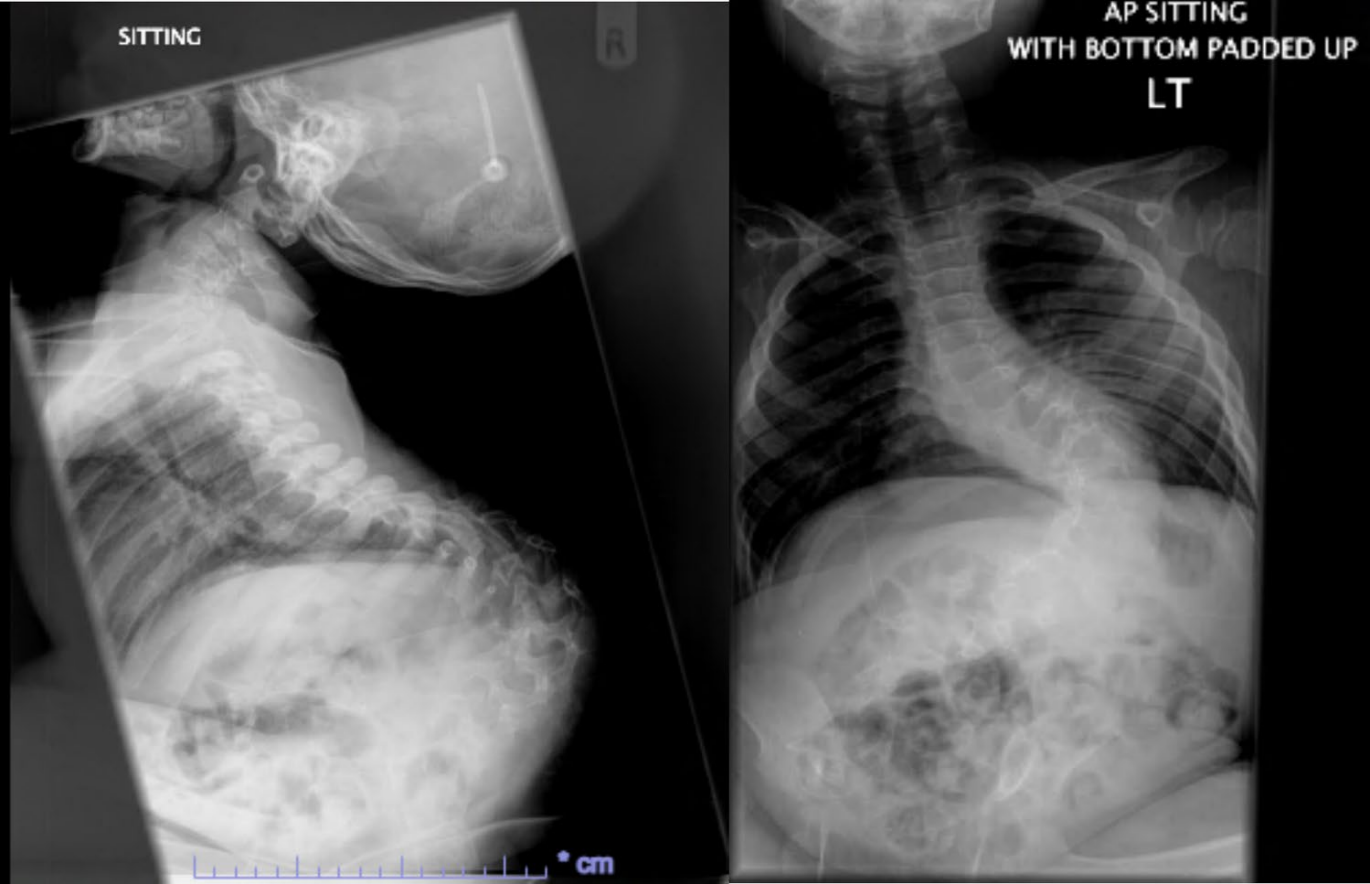
Scoliogram at referral; showing a left thoracolumbar curve of 100 degrees with a kyphotic deformity of 100 degrees

**Fig. 2:**
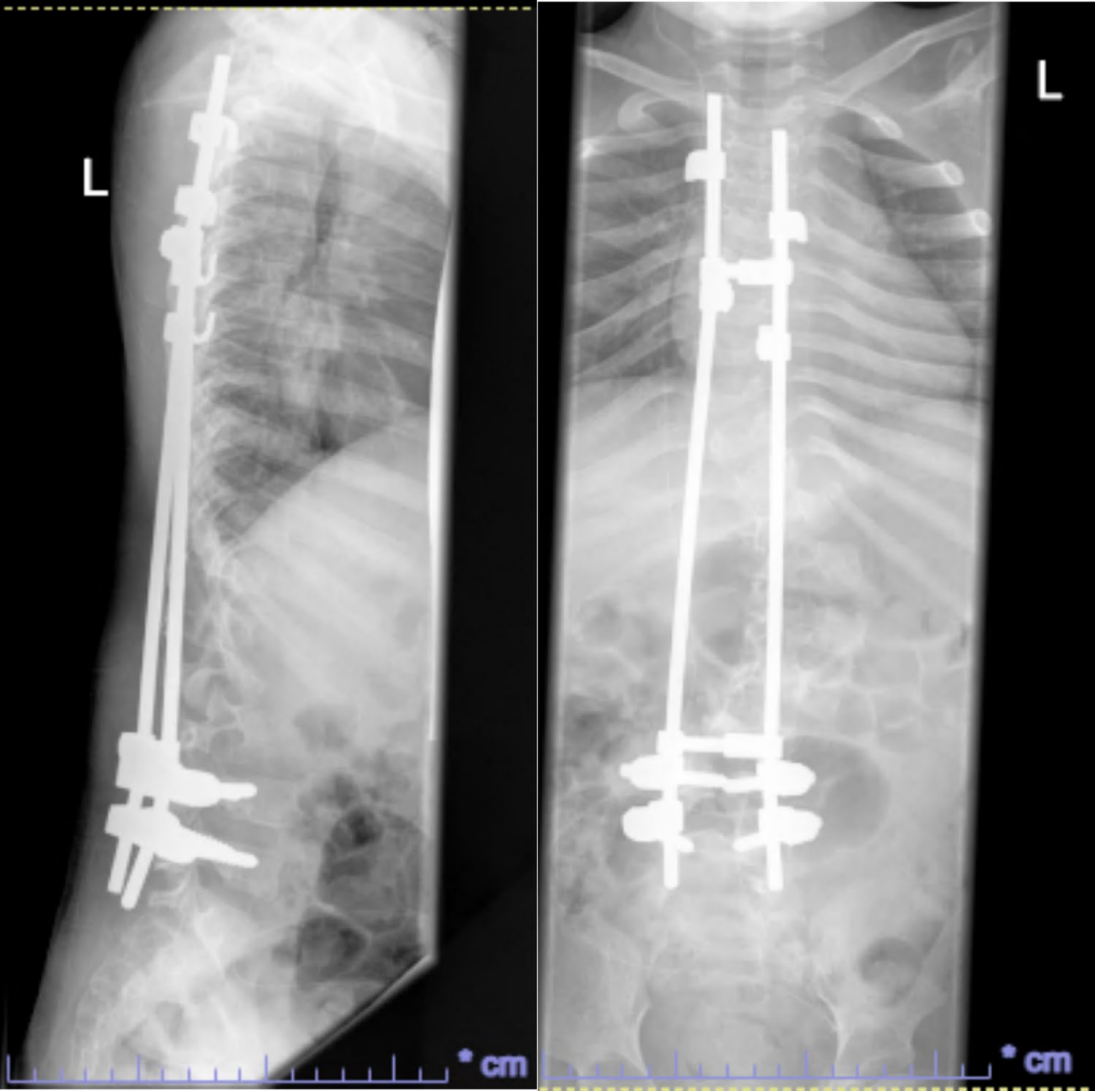
Post operative X-ray after implantation of TGR, aged 5

**Fig. 3:**
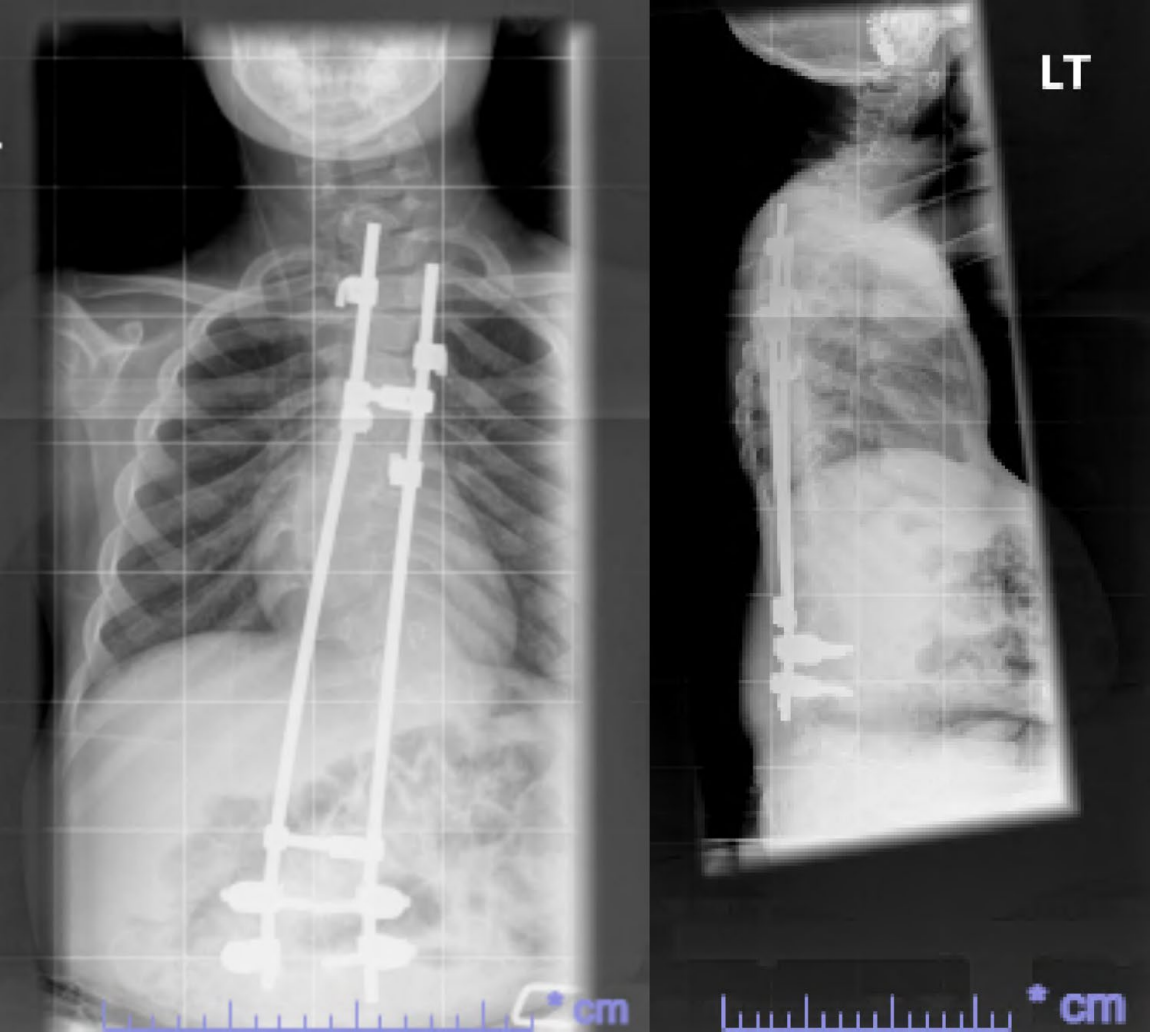
Post operative X-ray after first lengthening of TGR, aged 7

**Fig. 4:**
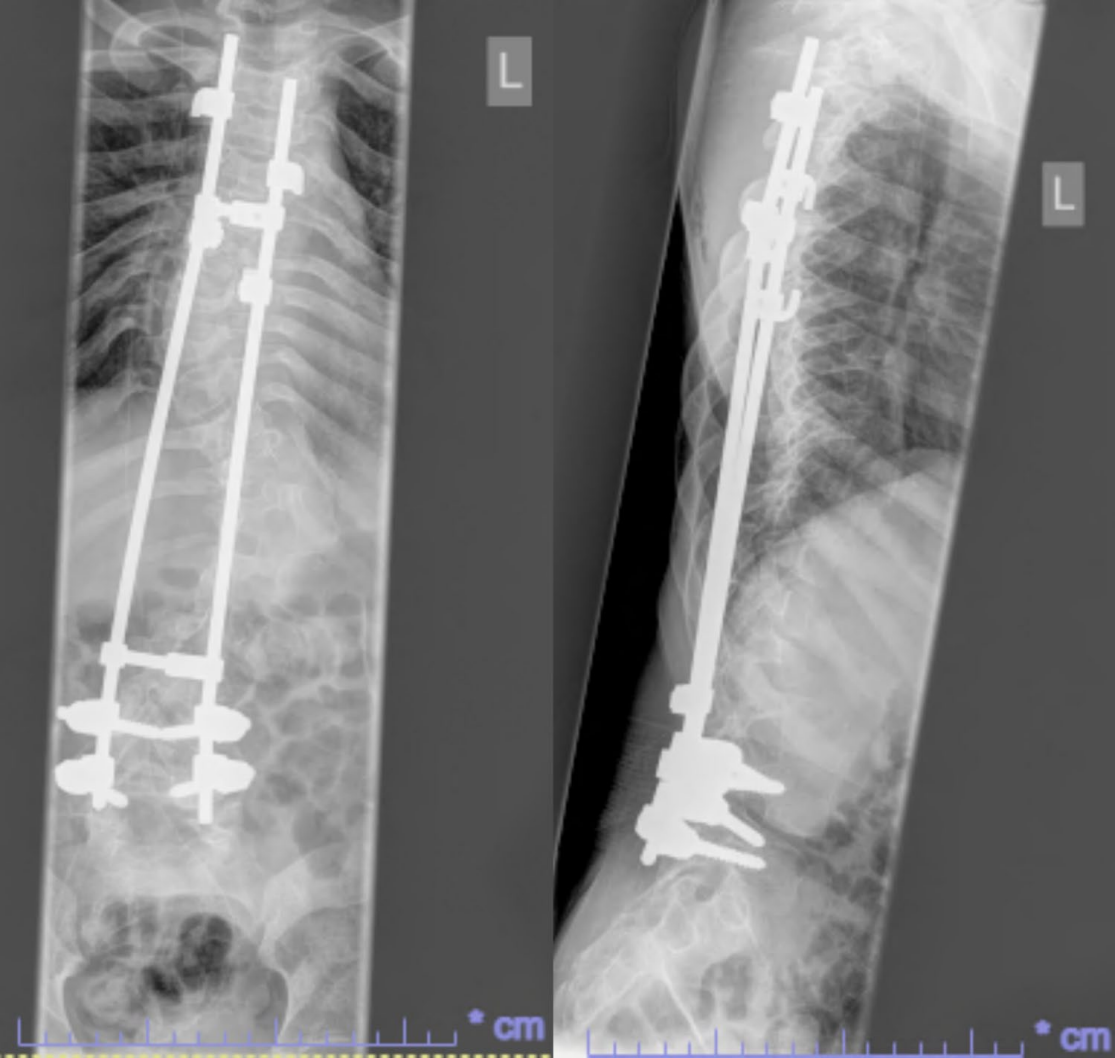
Post operative X-ray after second lengthening of TGR, aged 8

**Fig. 5:**
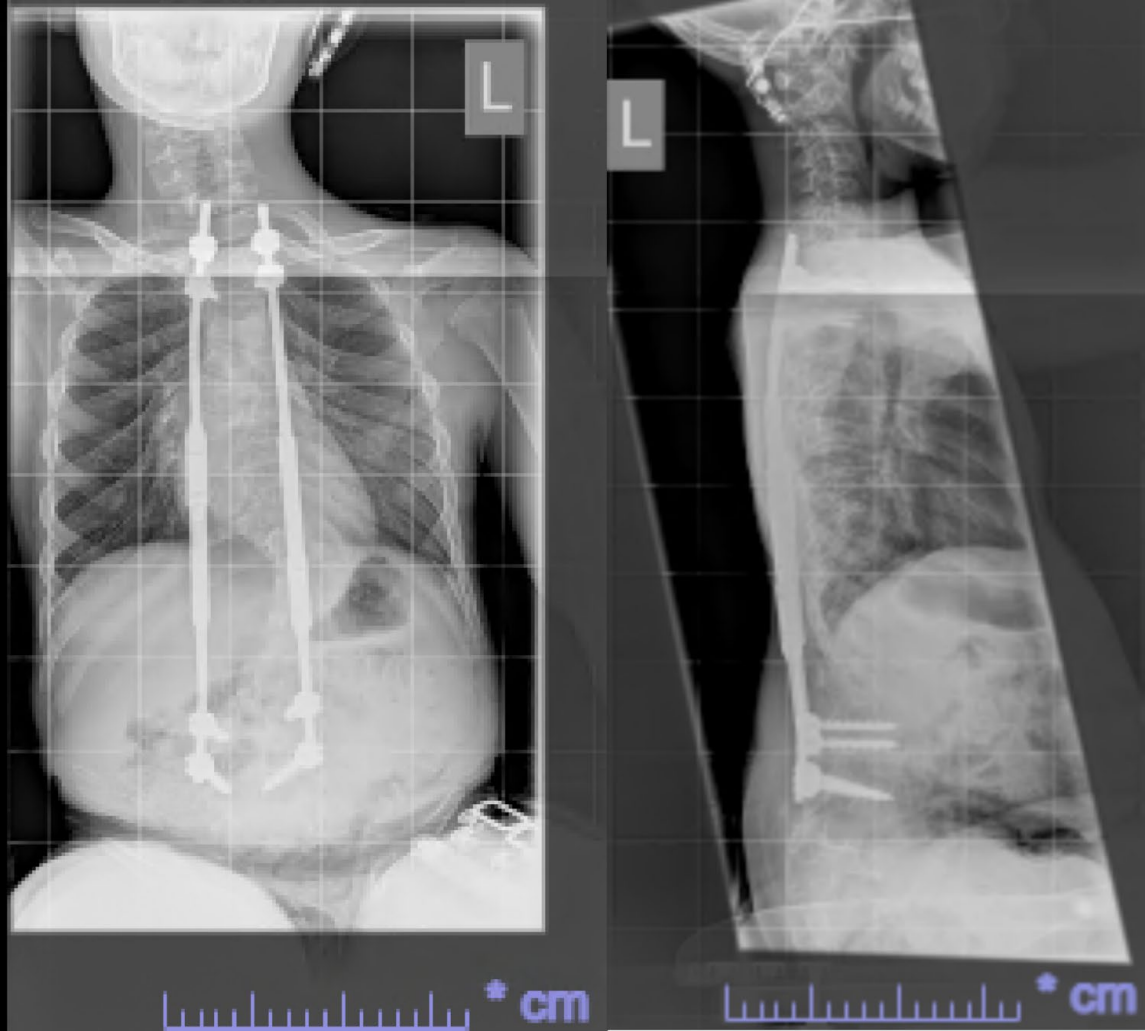
Post operative X-ray after implantation of MAGEC rods, aged 10

**Fig. 6:**
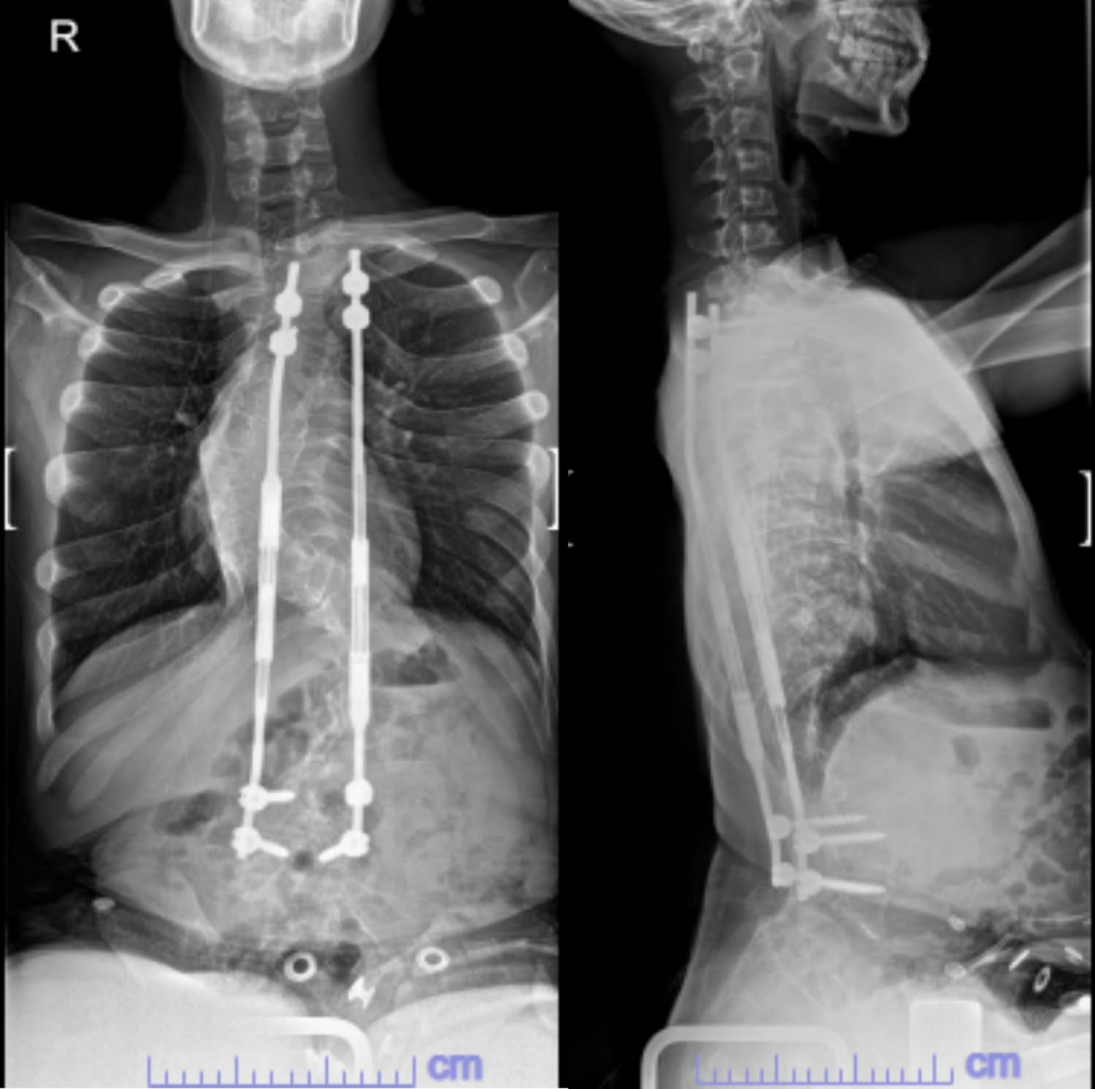
Most recent Scoliogram, aged 22

He had early onset kyphoscoliosis with curvature that was progressive and severe: by age 5 he had a left thoracolumbar curve of 100 degrees with a kyphotic deformity of 100 degrees (Figs. 1, 2, 3, 4, 5, 6).

He had traditional growing rods implanted at age 5. There was concern about bleeding risk due to the friability of his skin. His coagulation screen was normal, and hematology had recommended administering tranexamic acid and desmopressin acetate pre-operatively to minimize bleeding risk.

Due to his size and the friability of the skin, traditional dual growth rods with tandem connector were not used as its high profile risked skin breakdown. Instead, we used low-profile Mesa screws and rods (K2M Ltd). Two rods were connected, from T4 to L1, which were kept long proximally and distally, with the intention of achieving lengthening by utilizing the extra length of the rods above and below the screw foundations. All metalwork was covered by muscle or fascia and skin, and the wound was closed in layers. As the skin was extremely fragile, the procedure was performed with the help of a plastic surgeon.

There were no major complications; blood loss was 150 ml. An area of skin breakdown occurred intra-operatively when a disinfectant wipe was used to clean the skin prior to applying an electrode for spinal cord monitoring.

The growth rods were subsequently lengthened twice. After the first extension, 20 months after implantation, there was an increase of T1 to S1 height of 25 mm. A further extension was undertaken 1 year later.

With the introduction of the MAGnetic Expansion Control (MAGEC) rods in the UK, we believed that it would be the correct device to use for this patient. Although our patient did not experience any major complications with TGR, it would allow non-surgical lengthening, and thereby reduce the need for repeated surgeries and its implications on the skin and its healing potential.

Therefore, at 10 years old, the patient’s traditional growth rods were removed and replaced with dual MAGEC rods fixated from T2 to L4. K2M MESA pedicle screws were used again.

The procedure was complicated by 1000 cc of blood loss, attributed to the vascular fragility of this patient due to their aEDS. Immediately post-operatively, there was a superficial rash around the wound: upon changing the dressing, it healed within 3 weeks. A dermatologist reviewed the rash and made the diagnosis of allergic contact dermatitis.

The extensions were deliberately delayed by 6 months to allow the foundations of the growing rod construct to bed. Thereafter, extensions for the MAGEC rods were performed at 3 to 4 monthly intervals and monitored using X-rays initially, and ultrasound guided extensions subsequently. The rods were lengthened 13 times, an average extension of 3 mm each time.

No wound or metalwork complications occurred. The last extension of the MAGEC growing rods was performed when the patient was 16.5 years old. The patient was closely monitored for 2 years post-extensions without any issues or complications and subsequently, following discussions with the patient and family, the MAGEC rods were left in situ without definitive fusion.

He is currently in his 17th year of follow-up. His Cobb angle has remained stable 100 degrees with good correction of kyphosis. He still ambulates in a wheelchair (due to a chronic hip dislocation since aged 9) with good sitting balance.
